# Leachate management in medium- and small-sized sanitary landfills: a Greek case study

**DOI:** 10.1007/s11356-023-30934-6

**Published:** 2023-11-10

**Authors:** Olga P. Koutsou, Christoforos Mandylas, Michail S. Fountoulakis, Athanasios S. Stasinakis

**Affiliations:** Department of Environment, University of the Aegean, 81100 Mytilene, Greece

**Keywords:** Landfill leachate management, Biological treatment, Performance, Constructed wetlands, Evaporation ponds, Environmental footprint, GHG emissions

## Abstract

The sustainable management of landfill leachates remains a matter of important concern in many countries. We used as case study a medium-sized Greek landfill, and we initially investigated the performance of the existing secondary leachate treatment system. The activated sludge process removed chemical oxygen demand (COD), biochemical oxygen demand (BOD), NH_4_-N, and PO_4_-P by 55%, 84%, 94%, and 14%, respectively, but the effluents did not meet the legislation requirements for discharge or reuse. Afterwards, different management options of these effluents (co-treatment with sewage in the centralized treatment plant, onsite tertiary treatment with reverse osmosis, granular activated carbon (GAC), ozonation, photo-Fenton, or constructed wetlands) were evaluated regarding their operational costs and environmental footprint. The use of constructed wetlands presented the lower operational cost, energy requirements, and greenhouse gas (GHG) emissions, not exceeding 21.5 kg CO_2eq_/day. On the other hand, the power consumption and the GHG emissions of the other on-site technologies ranged from 0.37 kWh/m^3^ and 5.56 kg CO_2eq_/day (use of GAC) to 39.19 kWh/m^3^ and 588.6 kg CO_2eq_/day (use of ozonation), respectively. The co-treatment of the leachates with municipal wastewater required 0.6 kWh/m^3^ and emitted 30.18 kg CO_2eq_/day. For achieving zero-discharge of the treated leachates, a system consisting of constructed wetlands and evaporation ponds in series was designed.

**Supplementary Information:**

The online version contains supplementary material available at 10.1007/s11356-023-30934-6.

## Introduction

European Union countries (EU-27) aim to limit landfilling of solid waste to 10% of the total waste generated by 2035 (EU 2018). Despite the gradual decrease of the percentage of the solid wastes that are sent to the landfills through the years, 31.3% of the produced wastes in EU-27 were landfilled in 2020, while this practice was the prevailing method for several countries such as Greece, Finland, Sweden, Romania, and Bulgaria (Eurostat 2020; Katsouli and Stasinakis 2019). Specifically for Greece, 78.2% of the total produced wastes were landfilled in 2018, while 82 landfills were in operation by 2020 (ESDA 2020). Out of these, 29 are classified as medium-sized landfills serving 20,000 to 60,000 inhabitants, 15 are classified as small landfills (5000–20,000 inhabitants), and 25 are classified as very small landfills (<5000 inhabitants). The latter are located on Greek islands of the Aegean Sea.

Solid waste landfilling results to the daily production of important volumes of leachates (Podlasek 2023) that are characterized by intense color, high concentrations of organic compounds and ammonia nitrogen, existence of heavy metals, organic micropollutants, and microplastics (Nika et al. 2020; Mojiri et al. 2021; Narevski et al. 2021). Their characteristics are affected by several factors, such as the age of the landfill, the type and composition of the waste, the climatic conditions in the area, and the waste degradation rate in the landfill (Renou et al. 2008; Luo et al. 2020).

In Greece, the prevalent approach for managing leachates in nearly all landfills constructed within the past two decades involved on-site aerobic biological treatment using the activated sludge process and partial recirculation of the treated leachates within the landfill. The residual treated leachates are discharged into the aquatic environment or reused following the requirements of the Directive 91/271/EU and the Greek Regulation for Wastewater Reuse, respectively (EU 1991; YPEKA 2011). However, the experience from the operation of these landfill leachates treatment plants (LLTPs) has shown that factors such as the high initial chemical oxygen demand (COD) concentrations, the relatively low biochemical oxygen demand (BOD) to COD ratio, and the existence of toxic compounds that potentially inhibit microbial activity contribute to their inadequate performance (Remmas et al. 2018, 2022) and to the recording of terrestrial and aquatic pollution around Greece (Fatta et al. 1999; Elhag and Bahrawi 2016). As a result, the addition of tertiary treatment processes or/and the adoption of different management practices are required to assure environmental protection and human health.

Under this frame, several alternative options for the management of the secondary treated landfill leachates are reported to the literature (Özdemir et al. 2020; Reshadi et al. 2021), such as their transfer to centralized wastewater treatment plants (WWTPs) (Brennan et al. 2017a, 2017b), and their tertiary on-site treatment using reverse osmosis (RO) (Chen et al. 2021; Tsompanoglou et al. 2023), granular activated carbon (GAC) (Oloibiri et al. 2017; Mojiri et al. 2021), advanced oxidation processes (AOPs) (Silva et al. 2017; Gomes et al. 2019), or constructed wetlands (CWs) (Coppini et al. 2019; Silvestrini et al. 2019). The decision for the adoption of the optimal management method should be site-specific as it depends on several factors such as the treatment cost, the power requirements, the greenhouse gases (GHGs) emissions, the required staff workload, and the production of residues. For the smaller landfills that are monitored by limited personnel, the low operational costs and the simplicity of the selected processes are important issues for assuring the efficient operation of the LLTP. Additionally, the adoption of zero-discharge leachate solutions in these landfills protect the environment from potential failures of the equipment as the years progress. Despite the importance of the topic, so far, there are few available studies that compare different practices for the proper management of landfill leachates produced in small- and medium-sized landfills (Schiopu and Gavrilescu 2010; Smol and Generowicz 2018). These studies usually contain information for the technical characteristics of the different processes, but they do not take into account their environmental footprint. On the other hand, various factors, including the production of hazardous by-products (Gomes et al. 2019), energy consumption (Zhang et al. 2020), and GHG emissions (Tsompanoglou et al. 2023), play a significant role in selecting a suitable method for sustainable leachate management.

Based to the above, the main objective of the current article was to evaluate different technologies and practices for achieving sustainable management of secondary treated leachates of medium- and small-sized landfills. For this reason, a medium-sized landfill located in a Greek island was selected as case-study. Initially, the operation of the existing LLTP was monitored during a year in order to evaluate its performance and to systematically characterize the produced secondary effluents. Afterwards, the different management options of these effluents (co-treatment with municipal wastewater in the centralized WWTP, onsite tertiary treatment with RO, GAC, AOPs, or CWs) were evaluated for these secondary effluents taking into account the energy consumption, the GHG emissions, the operational cost, the produced residues, the area required, and the required workload. At the last part of the study, a zero-discharge system consisting of CWs and evaporation ponds in series was designed for the secondary treated landfill leachate management.

## Materials and methods

### Description of the studied Greek landfill and the existing LLTP

The studied sanitary landfill is located in a Greek island serving a population of 50,500 permanent inhabitants (coordinates: 38°15′46.6″N, 26°00′15.1″E). Its total available area is equal to 86,000 m^2^, where the landfill basin covers an area of 51,000 m^2^. The landfill commenced operations in October 2012, and by February 2019, it had received a total of 188,000 m^3^ of municipal solid waste (MSW). After compression, the solid waste was covered with 20-cm-thick soil on a daily basis. The composition of the MSW were 44% food waste, 22.2% paper and paperboard, 13.9% plastics, 4.3% glass, 3.9% metals, 4.6% wood, leather and textiles, and 6.8% other materials.

The existing LLTP consists of a storage tank of the raw leachates, an activated sludge system with two aerated activated sludge bioreactors in series, a third one operating under anoxic conditions and a settling tank with sludge recirculation, a chlorination tank, and a tank for the storage of the final effluents. During the period of study (February 2020 to February 2021), the average daily flow of leachates was approximately 25 m^3^/day. To improve the performance of the system, the activated sludge recirculation rate increased from 10 to 50 m^3^/day in March 2020, while the operation of diffusers in the aerobic reactors was adjusted in order to achieve a dissolved oxygen concentration higher than 2.5 mg/L. To improve the denitrification of produced nitrates, a flowrate of 10 m^3^/day of raw leachates was introduced to the anoxic bioreactor in September 2020. No pH adjustment of the raw leachates was conducted during the monitoring period.

### Monitoring and analysis of the quality of the raw and secondary treated leachates

#### Sampling

A total of 80 grab samples were collected from the inlet of the LLTP (raw leachates storage tank) and the outlet of the settling tank in 11 monthly sampling campaigns carried out between February 2020 and February 2021. In each sampling campaign, samples were collected for three or four consecutive days and they were sent to the lab under cooling conditions using portable Styrofoam coolers. After arriving to the lab, samples were measured for pH, conductivity, total suspended solids (TSS), and BOD, while measurements of COD, ammonium nitrogen (NH_4_-N), nitrate nitrogen (NO_3_-N), and phosphorus (PO_4_-P) were carried out during the next 7 days. Microsoft Excel was used for data processing and statistical analysis.

#### Chemical analyses

The measurements of all parameters were conducted according to the Standard Methods for Water and Wastewater Analysis (APHA 2005). A Consort C932 electrochemical analyzer and a Hach Sension 5 portable instrument were used for pH and electrical conductivity, respectively. COD was determined spectrophotometrically, NH_4_-N by titration after distillation of the samples, NO_3_-N by the cadmium reduction method, PO_4_-P with the ascorbic acid method, and BOD with the manometric method. Filters with a diameter of 47 mm and a porosity of 1 μm were used for TSS measurement and for the filtration of samples before analysis of NH_4_-N, NO_3_-N, and PO_4_-P.

### Description of the methodology applied for the evaluation of the tertiary leachate treatment technologies

The alternative options for the tertiary treatment of the secondary treated leachates which were studied in the current article were the transport and discharge of the treated effluents of the LLTP to the centralized WWTP, and their on-site tertiary treatment applying RO, GAC, ozonation, photo-Fenton, or CWs. The above methods were evaluated in terms of the area required, the staff workload for the operation and monitoring of the process, the requirement of an additional treatment step before and/or after the proposed technology (Gomes et al. 2020), the expected effluent quality (Gomes et al. 2019), the production of by-products (Gomes et al. 2019), the operational cost of the basic technology (Chen et al. 2021; Oloibiri et al. 2017; Gomes et al. 2020; Gomes et al. 2019), the energy consumption (Holloway et al. 2016; Tow et al. 2021), and the GHG emissions (Tsompanoglou et al. 2023). The GHG emissions from the application of RO, GAC, ozonation, and photo-Fenton were estimated taking into account the energy consumption of each technologies (Table S1). Information on the energy consumption *E* (kWh/m^3^ of treated wastewater) and the treatment cost (€/m^3^ of treated wastewater) of each of the tertiary treatment process was retrieved by four (4) scientific articles published between 2016 and 2021 (Table S1) (Gomes et al. 2019; Gomes et al. 2020; Holloway et al. 2016; Tow et al. 2021). Regarding the GHG emitted from free water surface flow (FWS) and horizontal subsurface flow (HSSF) CWs, these were based on articles referred in the review paper of Mander et al. (2014). Specifically, for FWS CWs, data for CO_2_, CH_4_, and N_2_O emissions (flux, area, flowrate) were available in three (3) articles, while the relevant information for the HSSF-CWs were available in seven articles (Table S2). Furthermore, the GHG emissions from the treatment of the secondary treated leachates in the centralized WWTP were estimated following the methodology described by Koutsou et al. (2018). The specific equations used for the estimation of GHG emissions of the different processes can be found in Section 1 of the Supplementary Material.

### Design of a CWs — evaporation pond system for zero-discharge landfill leachate management

#### Design of the CW

At the last part of the study, FWS and HSSF CWs were designed in series using an average influent flowrate (*Q*_in_) of 25 m^3^/day for the tertiary treatment of the landfill leachates. The flowrate of CW effluents (*Q*_out_) was calculated using the following equation:

1 $${Q}_{\textrm{out}}={Q}_{\textrm{in}}-{ET}_c\times A+P\times \textrm{A}$$

where *ET*_*C*_ is the average annual evapotranspiration (m/d), *P* is the average annual precipitation (m/day), and *A* is the area of the CW (m^2^).

The evapotranspiration (*ET*_*C*_) in the CW is given by:

2 $${ET}_c={K}_c\times {ET}_o$$

where *K*_*c*_ is the crop factor and *ET*_*O*_ is the reference evapotranspiration.

According to the literature, *K*_*c*_ depends on factors such as the type of plants in the CW, the season, the climatic conditions, and the characteristics of the wetland Djaman et al. 2018). Considering that the vegetation in CWs treating landfill leachates is limited, an average *K*_*c*_ value of 1.5 was considered in the current study. The reference evapotranspiration (*ET*_*O*_) in the wetland was estimated based on the FAO Pennman-Monteith equation:

3 $${ET}_o=\frac{0.408\varDelta \left({R}_n-G\right)+\gamma \left(\frac{900}{T}+273\right){u}_2\left({e}_s-{e}_a\right)}{\varDelta +\gamma \left(1+0.34{u}_2\right)}$$

where Δ is the slope of water vapor pressure curve (kPa/°C), *R*_*n*_ is the average net radiation flux density (MJ/m^2^ day), *G* is the ground heat flux density (MJ/m^2^ day), *γ* is psychrometric constant, *T* is the average temperature, *u*_2_ is the wind speed at height of 2 m (m/sec), *e*_*s*_ is the saturated water vapor pressure (kPa), and *e*_*a*_ is the actual water vapor pressure (kPa).

The design of the first stage CW (FWS) was made applying the methodology proposed by the task group of the International Water Association for the use of CWs (Dotro et al. 2017). Specifically, the design was based on the model of tanks in series, taking into account both the reduction of pollutants and background concentration (*p*-*k*-C* model).

4 $$A=\frac{PQ_i}{k_A}\left|{\left(\frac{C_i-{C}^{\ast }}{C_o-{C}^{\ast }}\right)}^{\frac{1}{P}}-1\right|$$

where *C*_*o*_ is the outflow concentration (mg/L), *C*_*i*_ is the inflow concentration (mg/L), C^*^ is the background concentration (mg/L), *k*_*А*_ is the first-order factor (m/day), *Q* is the inflow volumetric flow rate (m^3^/day), *h* is the water depth (m), and *P* is the apparent number of tanks in series.

The design of the seconds stage CW (HSSF) was also done applying the methodology reported above for achieving final effluents polishing.

#### Design of the evaporation pond

In the current study, two evaporation ponds were designed for the final discharge of the landfill leachates. For the design, data on the flowrates of leachates from the outlet of CWs, the precipitation that will end up directly in the ponds, and the evaporation from them were taken into account.

The monthly and annual evaporation from the ponds were calculated taking into account the meteorological data of Table S3 and applying three different calculation methods, proposed in the relevant literature (Potts 1988). Specifically, the following equations were used:

5 $${ET}_o=p\ \left(0.457\ {T}_{\textrm{mean}}+8.128\right)$$

(Blanney-Criddle 1950)

where *ET*_*o*_ is the evaporation (mm/day), *T*_mean_ is the mean monthly temperature (°C), and *p* is the mean daily percentage of daylight hours.

6 $${E}_{\textrm{lake}}=\left[\exp \left(\left(T-212\right)\left(0.1024-0.01066\ \ln \textrm{GLOBAL}\right)\right)-0.0001+0.0105\ \left({e}_s-{e}_a\right)\ 0.88\ \left(0.37+0.0041\ {u}_p\right)\right]\times {\left[0.04686\ {\left(0.0041\ T+0.676\right)}^7+0.01497\right]}^{-1}$$

(Lamoreaux/Kohler 1962)

where *E*_lake_ is the evaporation (inches/day), *T* is the mean monthly temperature (°F), GLOBAL is the global radiation (langleys/day), *e*_*s*_ is the water surface vapor pressure (inches Hg), *e*_*a*_ is the air vapor pressure (inches Hg), and *u*_*p*_ is the wind speed (mph)

7 $${E}_o=\left[\left(\left(550\ {T}_m\right)/\left(100-A\right)\right)+15\ \left(T-{T}_d\right)\right]/\left(80-T\right)$$

(Penman/Linacre 1977)

where *E*_*o*_ is the evaporation (mm/day), *T*_*m*_ is the mean monthly temperature corrected for area altitude (°C), *A* is the latitude (degrees), *T*_*d*_ is the dew point (°C), and *T* is the mean monthly temperature (°C).

The most conservative value calculated from the above equations was chosen for the design of the evaporation ponds. For calculating the technical characteristics of the evaporation ponds, a water mass balance was applied (Tchobanoglous et al. 2003). Specifically, it was considered that the sum of the annual inlet of leachates and the annual inlet of precipitates on the surface of the ponds was equal to the annual outlet of water through evaporation from the surface of the ponds.

## Results and discussion

### Quality characteristics of raw and secondary treated leachates

The average, maximum, and minimum concentrations of the main pollutants in the raw and treated leachates of the studied LLTP, as well as the outflow requirements for wastewater discharge to the aquatic environment or wastewater reuse for agricultural irrigation, are presented to Table 1.

**Table 1:** Chemical characteristics of the raw and secondary treated leachates in the studied landfill leachate treatment plant and limit values included in EU legislation for wastewater discharge to the aquatic environment and wastewater reuse in agriculture. Values are given as mean value ± standard deviation; the minimum and maximum values are reported in parentheses (number of samples per sampling point = 40)

Parameter	Raw leachates	Secondary treated leachates	EU Directive 91/271 for discharge to the aquatic environment	Greek legislation 145116/2011 for limited irrigation
pH	8.34 ± 0.22 (7.65–8.65)	8.21 ± 0.34 (7.33–9.05)	6.5–8.5	6.5–8.5
Conductivity (mS/cm)	12.12 ± 0.38 (6.80–15.70)	9.16 ± 1.56 (5.68–11.07)	-	-
TSS (mg/L)	55 ± 52 (8–285)	32 ± 29 (4–120)	≤ 35	≤ 35
COD (mg/L)	3,585 ± 962 (2,272–5,885)	1,583 ± 508 (799–2,898)	≤ 125	-
BOD (mg/L)	254 ± 83 (123–439)	47 ± 51 (0–165)	≤ 25	≤ 25
BOD/COD	0.08 ± 0.04 (0.02–0.18)	0.03 ± 0.04 (0.00–0.11)	-	-
NH_4_-N (mg/L)	722 ± 157 (283–974)	35 ± 66 (1–340)	≤ 15 (as ΤΝ)	<45 or <15 (as TN)^(a)^
NO_3_-N (mg/L)	1.0 ± 2.5 (0.0–11.0)	79.7 ± 47.7 (3.0–221.0)		
PO_4_-P (mg/L)	8.2 ± 4.1 (0.2–16.9)	10.7 ± 6.7 (2.5–22.2)	-	-

^(a)^For areas polluted with nitrates

According to the literature, the characteristics of the produced landfill leachates are affected by several factors, such as the landfill age, the type, composition and degree of degradation of the waste, and the climatic conditions in the area (Renou et al. 2008; Luo et al. 2020). Among the above factors, the age of the landfill is considered as the most critical factor, as its increase contributes to the gradual decrease of BOD concentrations and the BOD/COD ratio of the leachates. Specifically, high BOD/COD ratios (0.5–0.8) are observed in leachates originated from recently constructed landfills, while BOD/COD ratios less than 0.5 are found in leachates from intermediate and/or older landfills where an important part of the organic compounds has been degraded (Luo et al. 2020). At the same time, the pH values of the produced leachates are expected to increase with the age of the landfill, while on the contrary, NH_4_-N concentrations do not seem to be significantly affected by landfill age (Tchobanoglous and Kreith 2002; Deng and Englehardt 2007; Luo et al. 2020).

The characteristics of the raw leachates found in the current study are similar to those reported in the literature for landfills that receive municipal solid waste and characterized as intermediate in age (Tchobanoglous and Kreith 2002; Renou et al. 2008; Mohammad-Pajooh et al. 2017; Nika et al. 2020; Luo et al. 2020). Specifically, the mean value of pH and conductivity was 8.34 and 12.12 mS/cm, respectively, while the average concentrations of COD and NH_4_-N were 3585 mg/L and 722 mg/L, respectively (Table 1). On the other hand, the average concentration of BOD was significantly lower (254 mg/L), indicating that a small part of the organic load is biodegradable (BOD/COD ratio = 0.08), while the concentrations of TSS, NO_3_-N, and PO_4_-P were also relatively low. Some differences on the characteristics of raw leachates were observed during the different sampling months. Specifically, the highest COD values were observed during September, October, and November, where the average monthly concentration was greater than 4000 mg/L, while the highest values of NH_4_-N were observed in August, September, October, and November, where the average monthly concentration was greater than 800 mg/L (Fig. S1, S2).

Regarding the quality characteristics of the secondary treated leachates, the mean values of pH and conductivity were 8.21 and 9.16 mS/cm, respectively, while the average concentrations of COD and BOD exceeded the effluent requirements for wastewater discharge to the aquatic environment or wastewater reuse for irrigation, reaching 1583 mg/L and 47 mg/L, respectively (Table 1). The average concentration of TSS was marginally lower than the effluent requirements, while the average concentrations of nutrients were equal to 35 mg/L, 79.7 mg/L, and 10.7 mg /L for NH_4_-N, NO_3_-N, and PO_4_-P, respectively.

**Fig. 1:**
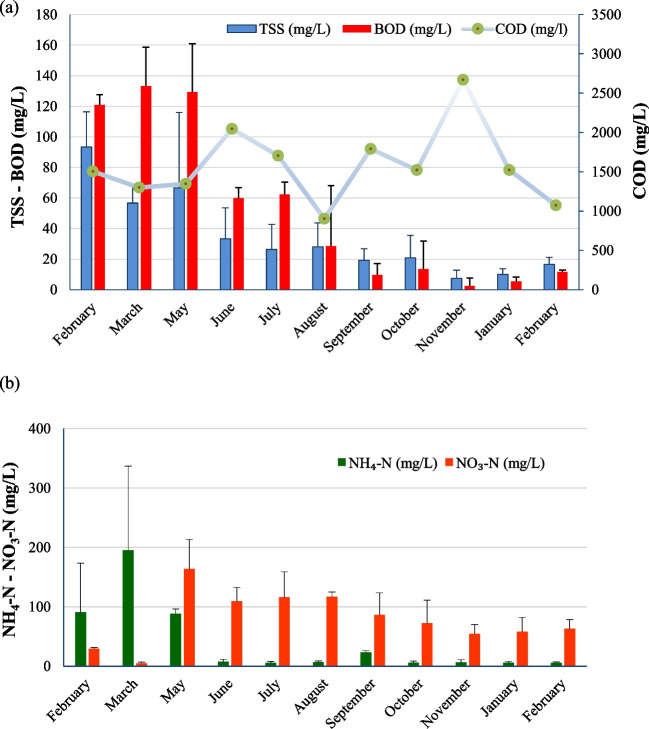
Average monthly concentrations of TSS, BOD, and COD (**a**) and NH_4_-N and NO_3_-N (**b**) in the secondary treated leachates of the studied landfill leachate treatment plant. The standard errors are also provided in Fig. 1b

Regarding the time variation of the parameters in the treated leachates, a gradual decrease in the concentrations of TSS and BOD was observed during the monitoring period (Fig. 1a). Specifically, from June 2020 until the end of monitoring period, the mean monthly TSS concentrations were lower than 35 mg/L, while from September 2020 and until the end of the monitoring period, the mean monthly BOD concentrations were lower than 25 mg/L, achieving for both parameters the limits for wastewater discharge and reuse. On the other hand, a similar trend in COD concentrations was not noticed and values higher than 850 mg/L were determined during the whole period (Fig. 1a).

Regarding NH_4_-N, its concentration decreased significantly after the first months due to improved nitrification achieved after the increase of sludge recirculation rate and the adjustment of DO concentrations mentioned in “Description of the studied Greek landfill and the existing LLTP” (Fig. 1b). As a result, between June 2020 and February 2021, the average monthly concentrations of NH_4_-N were lower than 10 mg/L (with the exception of September 2020 where its average concentration was 23 mg/L) (Fig. 1b). At the same time, after May 2020, the concentration of NO_3_-N increased significantly. The daily transfer of 10 m^3^/day of raw leachates in the anoxic bioreactor after September 2020 improved denitrification process resulting to the gradual reduction of NO_3_-N to concentrations lower than 70 mg/L (Fig. 1b).

### Performance of landfill leachates treatment plant

**Fig. 2:**
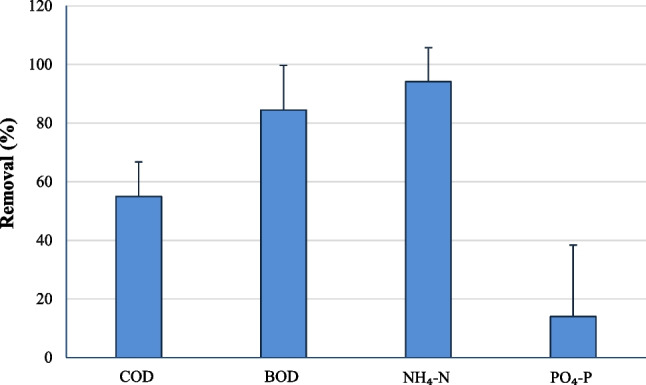
Average removal (%) of the main pollutants in the studied landfill leachates treatment plant (LLTP) (monitoring period 24.2.2020–23.2.2021). The standard errors are also provided

Calculation of the average removals of the main pollutants during the monitoring period shows that the studied LLTP achieved partial COD removal (55 ± 12%), high BOD removal (84 ± 15%), high NH_4_-N removal (94 ± 12%), and low PO_4_-P removal (14%) (Fig. 2).

Concerning possible changes to the performance of the system during the monitoring period, BOD and NH_4_-N removal rates were increased due to the corrective actions described in “Description of the studied Greek landfill and the existing LLTP” (Fig. S3, S4). Specifically, the removal of BOD during the first months of the monitoring period was in the range of 58 to 66% and it was gradually increased exceeding 93% after October 2020 (Fig. S3). The removal of NH_4_-N ranged between 69 and 86% during the first trimester of the monitoring period, whereas it was higher than 98% up to the end of the study period (Fig. S4). In contrast to the aforementioned parameters, a slight improvement on COD removal was gradually observed ranging up to 69% (Fig. S3).

The observed removals of COD, BOD, and NH_4_-N are similar or higher than those reported in the literature for aerobic biological treatment systems which treat landfill leachates. In a recent paper published by members of our research team, the removal of COD and NH_4_-N in several Greek activated sludge LLTP was 62% and 71%, respectively (Nika et al. 2020). In other previous articles, as shown in Table S4, the removals of COD, BOD, and NH_4_-N in various aerobic biological LLTP ranged between 40–79%, 61–99%, and 60–99%, respectively (Hoilijoki et al. 2000; Frascari et al. 2004; Neczaj et al. 2005; Visvanathan et al. 2007; Aluko and Sridhar 2013; Torretta et al. 2017; Tsompanoglou et al. 2023).

### Evaluation of different alternative technologies for tertiary landfill leachate treatment

Taking into account that the studied LLTP could not achieve the limits of the legislation for wastewater discharge or reuse, the co-treatment of secondary treated landfill leachates with municipal wastewater at the centralized WWTP, and their onsite tertiary treatment with RO, GAC, ozonation, photo-Fenton, or CWs were evaluated. The results of the evaluation for the area required, the staff workload, the need for additional treatment step before and/or after the proposed technology, the expected effluent quality, the production of by-products, the operating cost of the basic technology, the energy consumption, and the GHG emissions are presented in Table 2.

**Table 2:** Comparison of alternative management options for tertiary treatment of landfill leachates

Method	On-site area required	Staff workload	Need for additional treatment step before and/or after the proposed technology	Expected effluent quality	Production of by-products	Energy consumption (kWh/m^3^)	GHG emissions (kg CO_2eq_/d)	Operational cost of the technology (€/m^3^)	Annual operational cost (€/year)
Transport to centralized WWTP	No	No	No	Suitable for irrigation or discharge to the environment (COD < 125 mg/L, BOD < 25 mg/L, TSS < 35 mg/L)	Sewage sludge production	0.6^1^	30.2	4	18,200
Reverse osmosis	Small (<100 m^2^)	Medium (daily)	Filtration with a sand filter	Suitable for irrigation or discharge to the environment (COD < 10 mg/L, BOD < 5 mg/L, TSS < 5 mg/L)	Production of particularly burdened condensate (17-25% supply)	0.46^2^–0.56^3^	6.91–8.41	0.98–4.11^4^	8940–37,500
Granular activated carbon	Small (<100 m^2^)	Medium (daily)	Filtration with a sand filter or flocculation	Suitable for irrigation or discharge to the environment (COD < 70 mg/L, BOD < 10 mg/L, TSS < 5 mg/L)	Obligation of periodic regeneration of saturated activated carbon	0.37^3^	5.56	1.32^5^	12,045
Ozonation	Small (<100 m^2^)	Medium (daily)	Sand filter filtration and biological treatment as final stage	Suitable for irrigation or discharge to the environment (COD < 125 mg/L, BOD < 10 mg/L, TSS < 5 mg/L)	-	39.19^6^	58 8.6	6.0^7^	54,750
Photo-Fenton	Small (<100 m^2^)	Medium (daily)	Flocculation and biological treatment as final stage	Suitable for irrigation or discharge to the environment^8^ (COD < 125 mg/L, BOD < 10 mg/L, TSS < 5 mg/L)	Sludge production^8^	7.58^6^	113.9	2.5–5.1^8^	22,800–46,500
Constructed wetlands	Medium (2.000–3.000 m^2^)	Low (once a week)	Disposal in evaporation pond as final stage	Not suitable for irrigation or discharge to the environment (COD < 250 mg/L, BOD < 25 mg/L, TSS < 35 mg/L)	-	-	12.55 (FWS)^9^, 8.78 (HSSF)^9^	0.1	915

^1^Current study

^2^Holloway et al. (2016)

^3^Tow et al. (2021)

^4^Chen et al. (2021)

^5^Oloibiri et al. (2017)

^6^Information taken from Table S1

^7^Gomes et al. (2020)

^8^Gomes et al. 2019

^9^Information taken from Table S2

The transport of the secondary treated leachates to the centralized WWTP has several advantages as does not require area on-site for the treatment of leachates and does not increase the workload of landfill’s staff. The primary challenge encountered by municipal WWTPs when receiving landfill leachates is the frequent occurrence of elevated NH_4_-N concentrations within the leachates. However, recent studies by the Irish Environmental Protection Agency showed that when the volumetric loading from leachates’ transport does not exceed 4% of the total daily charge of the WWTP and when the daily load of NH_4_-N of the leachates does not exceed 50% of the total daily incoming load to the WWTP, there is no observed hindrance to the nitrification process, nor any discernible impact on the system's performance (Brennan et al. 2016; Brennan et al. 2017a, 2017b). For the current case-study, the volumetric loading from leachate transport was calculated to 0.36%, significantly less than the limit of 4% reported in the literature (Brennan et al. 2016; Brennan et al. 2017a, 2017b) while the daily load of the leachates’ NH_4_-N was calculated to 0.875 kg/day, much lower than the 210 kg/day total load received by the centralized WWTP from municipal wastewater. The energy consumption and the GHG emissions of this scenario were calculated to 0.6 kWh/m^3^ of leachates and 30.18 kg CO_2eq_/day, respectively. Calculating the contribution of leachates’ transport and leachates’ treatment to the total GHG emissions, it can be seen that the transport emits 6.18 kg CO_2eq_/day while the treatment is responsible for the rest 24 kg CO_2eq_/day. Among different procedures related to leachates treatment in this WWTP, the highest amounts of GHGs are emitted due to GHG production from biomass decay and GHG production from net power consumption (Table 3). The yearly cost for the transport of leachates was estimated to 18,200 €.

**Table 3:** GHG emissions (kg CO_2eq_/d) from the co-treatment of treated leachates with municipal wastewater to the centralized WWTP

Procedure	GHG emission (kg CO_2eq_/d)
GHG emission from the treatment of leachates in WWTP	24
GHG production from biomass decay	9.70
GHG production from BOD removal and biomass production	1.59
GHG production from nitrification and denitrification	4.24
GHG consumption from nitrification	0.41
GHG production from sludge disposal to the landfill	7.20E-06
GHG production from net power consumption	8.88
GHG emission from the transport of the leachates to the WWTP by tanker	**6.18**
Total GHG emission	**30.18**

The on-site application of RO, GAC, ozonation, or photo-Fenton for the treatment of leachates results to low requirement of area for the installation of the reactors (<100 m^2^). Τhe work of the staff for the operation of these systems will be necessary in a daily-basis, while additional treatment steps are required before or/after the processes for assuring efficient performance (Table 2). A filtration step should be applied before RO and GAC, sand filtration, and biological treatment are usually required as polishing steps after ozonation, while flocculation and biological treatment are often used after photo-Fenton process. The quality of the effluents from the aforementioned tertiary treatment processes will fulfill the limit values of the legislation for wastewater discharge and reuse. Typically, COD, BOD, and TSS concentrations at the effluents of these tertiary treatment processes are lower than 125 mg/L, 10 mg/L, and 5 mg/L, respectively (Tchobanoglous et al. 2003), while the higher quality of the final outlet is achieved using RO (Table 2). Concerning the power consumption of these technologies, the values range from 0.37 kWh/m^3^ (use of GAC) to 39.19 kWh/m^3^ (use of ozonation). The application of RO required 0.46 to 0.56 kWh/m^3^ while the use of photo-Fenton 7.58 kWh/m^3^ (Table 2). The estimated GHG emissions ranged between 5.56 kg CO_2eq_/day (use of GAC) and 588.6 kg CO_2eq_/day (use of ozonation). High GHG emissions (113.9 kg CO_2eq_/day) were also calculated for photo-Fenton process. Regarding the yearly costs, the lower cost was calculated for the use of GAC (12,045 €), based to the article of Oloibiri et al. (2017) that reported a cost of 1.32 €/m^3^ for secondary treated landfill leachates. On the other hand, the highest cost was calculated for ozonation (54,750 €) using data from Gomes et al. (2020) who reported an operational cost of 6 €/m^3^ for secondary treated landfill leachates. Concerning the other tertiary treatment processes, Gomes et al. (2019) reported that the cost of photo-Fenton ranges between 2.5 and 5.1 €/m^3^, while Chen et al. 2021 calculated the cost of RO treatment between 0.98 and 4.11 €/m^3^. Among the different components contributing to RO operational cost, electricity consumption is the major followed by membranes replacement, chemical consumption, and maintenance fees (Chen et al. (2021). It should be mentioned that among these processes, the application of RO results to the formation of important amounts (17 to 35% of the inflow) of heavily contaminated retentate that needs additional treatment or/and it is recirculated in the landfill (Tsompanoglou et al. 2023). The conductivity in RO retentate can reach some tens of mS/cm, while the concentrations of COD and NH_4_-N exceed 2500 mg/L and 200 mg/L, respectively. Heavy metals are also detected at concentrations up to few mg/L (Chen et al. 2021). In the current study, it was assumed that the RO retentate will be recirculated to the landfill, and therefore, its contribution to the operational cost of the process was not calculated.

The treatment of leachates in FWS-HSSF CWs requires important areas for the construction of the systems (> 1000 m^2^). The exact required area will be presented in “Design of the FWS-HSSF constructed wetland and the evaporation pond.” Τhe workload of the staff for the operation of the systems will be limited, while no additional treatment steps are required. The energy consumption for the operation of these systems is negligible, and it is only due to the use of pumps for the transfer of leachates. Concerning the GHG emissions, they were estimated at 12.55 kg CO_2eq_/day for FWS and 8.78 kg CO_2eq_/day for HSSF CWs, respectively (Table 2). To perform these calculations, the GHG emission data reported by Søvik et al. (2006) was used (Table S2). This choice was influenced by the comparable scale of area and flowrate observed in their study, aligning with the corresponding characteristics of the CW examined in our current research. The yearly operational cost of leachate treatment was estimated to lower than 1000 €. Given the potential presence of persistent organic compounds in the effluents from the CWs, which could hinder achieving COD limit values, the final discharge of the effluents to an evaporation tank should be considered to assure environmental protection.

### Design of the FWS-HSSF constructed wetland and the evaporation pond

Taking into account the lower operational cost of CWs, the minimum staff workload, the negligible requirement for energy, and the lower GHG emissions, a CW consisting of FWS and HSSF in series was designed for the tertiary treatment of landfill leachates. The outlet of this system should be transferred to a pond for storage and evaporation. The methodology for the design of CWs has been described in “Design of the CW.” It was based on the desired BOD, NH_4_-N, and NO_3_-N concentrations at the effluents. The parameters selected for the HSSF and FWS design are presented in Table S5.

According to the results, it was calculated a total surface area of 1148 m^2^ for the HSSF. Constructing two similar CWs (length/width ratio = 3:1, bed depth = 0.5), the organic loading rate per flow surface will be equal to 128 g/m^2^ day which is lower enough than the maximum allowable organic loading per flow area (250 g/m^2^ day) (Dotro et al. 2017). The total surface area for the FWS was 925 m^2^. The construction of two similar CWs is also suggested (length/width ratio = 3:1, bed depth = 0.3). The average hydraulic residence time, HRT in HSSF and FWS, was calculated at 7.4 and 8.4 days, respectively. The design characteristics of the CWs are reported in detail in Table S6. Concerning the final outlet of the CWs, it was found that their average daily outflow will vary between 0 m^3^/day (June, July, and August) and 34.3 m^3^/day (December) (Table S7).

As regard to the evaporation ponds, in areas where the annual evaporation exceeds annual precipitation, evaporation of leachates in open ponds is a low-cost disposal method that is applied worldwide (Kim 2011; Amoatey et al. 2021). According to the Australian Environment Agency (EPA 2019), these ponds should be impermeable to prevent the contamination of the water table, their design, and construction should effectively block surface runoff entry, and their volume must be adequate to prevent overflow. Monitoring for leaks should be given due attention. To design two similar evaporation ponds, data was used from the average daily outflow of the wetlands (Table S7) as well as meteorological data for the calculation of the amounts of water entering the evaporation ponds due to precipitation (Table S3).

**Fig. 3:**
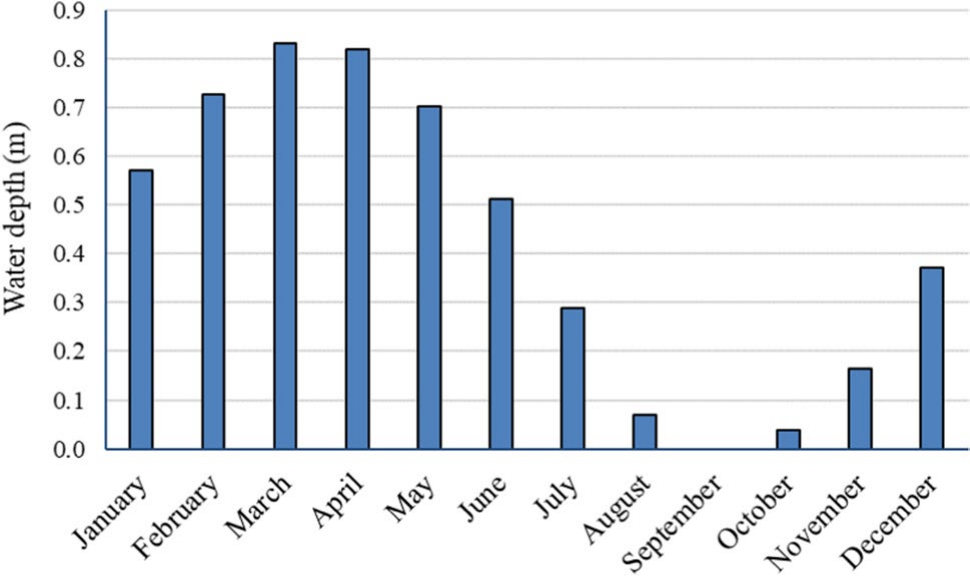
Monthly variation of the water depth in the evaporation ponds

The calculated annual evaporation values ranged between 1637 and 2026 mm/year. The most conservative value of 1637 mm/year was chosen for the design of the evaporation ponds. The above value also approaches the value of the annual evaporation (Penman potential evaporation, ETp) of 1528 mm/year, which has been reported in the literature for other islands of the North Aegean (Kitsara et al. 2009). The monthly inflows of water into the ponds due to leachates and precipitation, as well as the outflows due to evaporation are presented in Table S8. To reduce the required area of the evaporation ponds, the installation of an evaporator in one of the ponds is also suggested (Fig. S5). According to the technical characteristics of a commercial evaporator (3 HP, Kasco, 3.1EVFX), an extra quantity of liquid equal to 2.3 m^3^ per hour of operation can be evaporated. The operation of this system for 4 h a day and 7 months a year (November to May) will result in an additional evaporation of the liquid, equal to 1950 m^3^ per year. Based to these calculations, the total surface area needed for the evaporation ponds, where the yearly sum of inflows and outflows is balanced, was computed as 4,440 m^2^. According to the last column of Table S8 and Fig. 3, the maximum depth of the liquid will be 0.8 m (March, April), while during September and October, water will have been evaporated from the ponds.

## Conclusions

The monitoring of the existing LLTP for 1 year showed an average COD, BOD, NH_4_-N, and PO_4_-P removal of 55%, 84%, 94%, and 14%, respectively. The mean values of pH and conductivity in the secondary treated leachates were 8.21 and 9.16 mS/cm, respectively, while the relevant concentrations of COD, BOD, and TSS were 1583 mg/L, 47 mg/L, and 32 mg/L, exceeding for COD and BOD the effluent requirements for wastewater discharge or reuse. Among the different management options of secondary treated leachates, CWs presented the lower operating costs, required amounts of energy and GHGs emissions. Some of the limitations of this technology that allow their application only in small and medium-size landfills are the significantly larger area required than the other on-site technologies and the COD concentrations at the effluents than cannot meet the legislation limit value of 125 mg/L. For this reason, the construction of CWs should be combined with the construction of evaporation ponds, aiming to the zero discharge of the final effluents. Further research is required on the environmental footprint (GHG emissions, energy consumption) of the different tertiary treatment processes when applied in landfill leachates.

### Supplementary information

ESM 1 (DOCX 944 kb)
