# Supplementary material for: Leachate management in medium- and small-sized sanitary landfills: a Greek case study
Source: Environ Sci Pollut Res Int. 2023 Nov 10;30(57):120994–1006. doi: 10.1007/s11356-023-30934-6 (PMC10698084; doi:10.1007/s11356-023-30934-6)
Supplement: Supplementary file 1 — (DOCX 944 kb) [file 11356_2023_30934_MOESM1_ESM.docx]

**Leachate management in medium- and small-sized sanitary landfills: a Greek case study**

Olga P. Koutsou, Christoforos Mandylas, Michail S. Fountoulakis, Athanasios S. Stasinakis*

Department of Environment, University of the Aegean, 81100 Mytilene, Greece

The following tables and figures are included as supplementary information for this paper:

**Section 1:** Methodology applied for the estimation of GHG emissions by different tertiary leachate treatment process

**Number of supplementary Tables:** 9

**Number of supplementary Figures:** 5

**Section 1.** Methodology applied for the estimation of GHG emissions by different tertiary leachate treatment process

The estimation of GHG emissions (kg/d) (Table S1) from the application of RO, GAC, ozonation or photo-Fenton to the secondary treated leachates was conducted as described below:

The emitted amounts of GHG per amount of total electricity produced in Greece (expressed as kg/kWh (Table S9) were estimated by Equation 1:

$$GHG emission intensity of total electricity generation =\frac{GHG emissions}{electricity production}$$

(Eq. 1)

Where, GHG emissions are the emissions from electricity and heat production (produced by liquid, solid and gaseous fuels) for 2019 (Table S9) while the gross electricity production for this year was equal to 45.5 TWh (65.4% from the use of fossil fuels and 34.6% from renewable energy sources) (Greek Ministry of Environment and Energy 2021).

The GHG (CO_2_, CH_4_ and N_2_O) emissions (as kg/m^3^ of treated wastewater) (Table S1) from each process (RO, GAC, ozonation and photo-Fenton) was calculated using Equation 2, based on the above GHG emission intensity of total electricity generation (kg/kWh) and the energy consumption E (kWh/m^3^ of treated wastewater):

$GHG emissions= E*GHG emission intensity of total electricity generation$ (Eq. 2)

For the cases that the energy consumption, E (Table S1) was not given in the literature, it was estimated using Equation 3:

$E=\frac{Treatment cost}{Energy market price}$ (Eq. 3)

Where, the treatment cost is measured in €/m^3^ of treated wastewater and the energy market price is measured in €/kWh (Table S1).

The GHG (CO_2_, CH_4_ and N_2_O) emission values (as kg/m^3^ of treated wastewater) from the CWs (Table S2) were estimated by Equation 4:

$GHG emissions=\frac{J\times S}{Q}\times conversion factor$ (Eq. 4)

Where, J is the (N_2_O-N, CO_2_-C, CH_4_-C) flux (kg/m^2^d) from the wetland system (based on data retrieved by Mander et. al. (2014)), S is the wetland’s surface area (m^2^), and Q is the wastewater flowrate (m^3^/d). As conversion factors in Eq. 4, the value 44/28 was used for converting g N_2_O-N to g N_2_O (44 and 28 is the molecular weight of N_2_O and N_2_, respectively), the value 44/12 was used for converting kg CO_2_-C to kg CO_2_ (44 and 12 is the molecular weight of CO_2_ and C, respectively) and the value 16/12 was used for converting g CH_4_-C to g CH_4_ (16 and 12 is the molecular weight of CH_4_ and C, respectively).

For the aforementioned treatment options (use of RO, GAC, ozonation, photo-Fenton and CWs), GHG (CO_2_, CH_4_ and N_2_O) emissions were converted into kg/d (Table S2, S1) using Equation 5:

$GHG (kg/d) =GHG emission\times Q$ (Eq. 5)

Where, GHG emission is CO_2_, CH_4_ and N_2_O emissions respectively in kg/m^3^ and Q is the average daily flowrate of leachates (25 m^3^/day).

Since the global warming potentials of N_2_O and CH_4_ is equal to 298 and 28 times higher, respectively, than CO_2_ in the period of 100 years, GHG were expressed in units of CO_2eq_ (kg/d) (Table S2, S1) using Equation 6:

$CO_{2eq}={CO}_{2}+(28\times{CH}_{4})+(298\times N_{2}O)$ (Eq. 6)

Regarding the GHG emissions (g CO_2eq_/d) from the co-treatment of treated leachates to the centralized WWTP, the emissions from the transport of leachates and from their treatment were calculated separately. The GHG emissions from the transport of the leachates by tanker was estimated by Equation 7:

${CO}_{2eq}=\frac{Q}{V}\times2\times d\times a$ (Eq. 7)

Where, Q is the average daily flowrate of leachates (25 m^3^/day), V is the volume of the tanker (25 m^3^), d is the distance between landfill and centralized WWTP (19.3 km), and α is the GHG emission factor of the fuel (160 gCO_2eq_/km) (EEA 2019).

For the GHG emissions for leachates treatment, the energy consumption (kWh/m^3^) for their treatment in the centralized WWTP was initially estimated by Equation 8:

$Energy consumption=\frac{E_{required}}{Q}$ (Eq. 8)

Where, E_required_ (kWh/d) is the electricity consumption estimated by the methodology described in Koutsou et al. (2018) and Q is the average daily wastewater flowrate (m^3^/day).

Afterwards, the GHG emissions (g CO_2eq_/d) from leachates treatment in the centralized WWTP (Table 3) were estimated according to Koutsou et al. (2018). Specifically, it was taken into account the CO_2_ production from biomass decay, the CO_2_ production from BOD removal and biomass production, the CO_2_ consumption from nitrification, the CO_2_ production from denitrification, the N_2_O production from nitrification and denitrification processes, the net power consumption and the GHG emissions from the sludge disposal.

**REFERENCES**

EEA (2019) Average CO_2_ emissions from new cars and new vans. <https://www.eea.europa.eu/highlights/average-co2-emissions-from-new-cars-vans-2019>. Accessed 2022-11-27

Koutsou OP, Gatidou G, Stasinakis AS (2018) Domestic wastewater management in Greece: greenhouse gas emissions estimation at country scale. J Clean Prod 188: 851–859. <https://doi.org/10.1016/j.jclepro.2018.04.039>

Mander Ü, Dotro G, Ebie Y, Towprayoon S, Chiemchaisri C, Nogueira SF, Jamsranjav B, Kasak K, Truu J, Tournebize J, Mitsch WJ (2014) Greenhouse gas emission in constructed wetlands for wastewater treatment: A review, Ecol Eng 66: 19–35. <https://doi.org/10.1016/j.ecoleng.2013.12.006>

Ministry of Environment and Energy (2021) National Inventory Report of Greece for Greenhouse and Other Gases for the years 1990-2019. Greece. <https://ypen.gov.gr/wp-content/uploads/2021/06/2021_NIR_Greece.pdf>

1. **Supplementary Tables**

**Table S1** Treatment cost (€/m^3^ of treated wastewater), energy market price (€/KWh), energy consumption E (KWh/m^3^ of treated wastewater) and GHG emissions of each of the tertiary treatment process. The references used are given below.

| **According to the literature** | | | | **Estimated in the present study** | | | | | | | |
| --- | --- | --- | --- | --- | --- | --- | --- | --- | --- | --- | --- |
| **Process** | **Treatment (energy) cost** **€/m^3^** | **Energy market price (€/kWh )** | **Energy consumption (kWh/m^3^)** | **Energy consumption (kWh/m^3^)** | **CO_2_ (kg/m^3^)** | **CH_4_**  **(g/m^3^)** | **N_2_O (g/m^3^)** | **CO_2_ (kg/d)** | **CH_4_ (g/d)** | **N_2_O**  **(g/d)** | **CO_2eq_**  **(kg/d)** |
| Photo-Fenton^(Gomes et al. 2019)^  (Cost of multistage leachate treatment with PF stage as the major contributor) | 0.91^(Gomes et al. 2019)^  (mean value of all trials) (Calculated on the basis of the treated leachate volume,considering service time of lamps) | 0.12^(Gomes et al. 2019)^ |  | 7.583 | 4.542 | 0.0683 | 0.04163 | 113.56 | 1.71 | 1.04 | 113.9 |
| O_3_-only (with FluHelik/BC-Ventouri system^(Gomes et al. 2020)^ | 5.0^(Gomes et al. 2020)^ | 0.1276^(Gomes et al. 2020)^ |  | 39.185 | 23.472 | 0.3531 | 0.21513 | 586.79 | 8.83 | 5.38 | 588.6 |
| RO^(Holloway et al. 2016)^ |  |  | 0.46^(Holloway et al. 2016)^ |  | 0.276 | 0.0041 | 0.00253 | 6.89 | 0.10 | 0.06 | 6.91 |
| RO^(Tow et al. 2021)^ |  |  | 0.56^(Tow et al. 2021)^ |  | 0.335 | 0.0050 | 0.00307 | 8.39 | 0.13 | 0.08 | 8.41 |
| GAC-based advanced treatment plants in the US (including complementary treatment processes such as ozonation)^(Tow et al. 2021)^ |  |  | 0.37^(Tow et al. 2021)^ |  | 0.222 | 0.0033 | 0.00203 | 5.54 | 0.08 | 0.05 | 5.56 |

**REFERENCES**

1. Gomes AI, Foco MLR, Vieira E, Cassidy J, Silva TFCV, Fonseca A, Saraiva I, Boaventura RAR, Vilar VJP (2019) Multistage treatment technology for leachate from mature urban landfill: Full scale operation performance and challenges. Chem Eng J 376: 120573. <https://doi.org/10.1016/j.cej.2018.12.033>
2. Gomes AI, Soares TF, Silva TFCV, Boaventura RAR, Vilar VJP (2020) Ozone-driven processes for mature urban landfill leachate treatment: Organic matter degradation, biodegradability enhancement and treatment costs for different reactors configuration. Sci Total Environ 724: 138083. <https://doi.org/10.1016/j.scitotenv.2020.138083>
3. Holloway RW, Miller-Robbie L, Patel M, Stokes JR, Munakata-Marr J, Dadakis J, Cath TY (2016) Life-cycle assessment of two potable water reuse technologies: MF/RO/ UV–AOP treatment and hybrid osmotic membrane bioreactors. J Membr Sci [507](https://www.sciencedirect.com/journal/journal-of-membrane-science/vol/507/suppl/C):  165-178. <https://doi.org/10.1016/j.memsci.2016.01.045>
4. Tow EW, Hartman AL, Jaworowski A, Zucker I, Kum S, AzadiAghdam M, Blatchley III ER, Achilli A, Gu H, Urper GM, Warsinger DM (2021) Modeling the energy consumption of potable water reuse schemes. Water Res X 13: 100126. <https://doi.org/10.1016/j.wroa.2021.100126>

**Table S2** Estimated GHG emissions (as kg/m and kg/d) from the FWS and HSSF CWs designed for the tertiary treatment of landfill leachates.

|  |  | **CO_2_-C flux**  **(mg m^−2^ h^−1^)** | **CH_4_-C flux**  **(mg m^−2^ h^−1^)** | **N_2_O-N flux**  **(mg m^−2^ h^−1^)** | **CO_2_**  **(kg m^−3^)** | **CH_4_**  **(g m^−3^)** | **N_2_O**  **(g/m^3^)** | **CO_2_**  **(kg/d)** | **CH_4_**  **(kg/d)** | **N_2_O**  **(kg/d)** | **CO_2eq_**  **(kg/d)** |
| --- | --- | --- | --- | --- | --- | --- | --- | --- | --- | --- | --- |
| **FWS** | Full scale | 108.3^(f)^ | 8.4^(f)^ | 0.007^(f)^ | 0.353 | 9.946 | 0.0098 | 8.82 | 0.25 | 0.00024 | 15.85 |
|  |  | 95.8^(f)^ | 4.4^(f)^ | 0.106^(f)^ | 0.312 | 5.210 | 0.148 | 7.80 | 0.13 | 0.0037 | 12.55 |
|  |  | 100^(g)^ | 4.8^(g)^ | 0.23^(g)^ | 0.326 | 5.683 | 0.321 | 8.14 | 0.14 | 0.00802 | 14.51 |
|  |  | 29.4^(f)^ | 1.6^(f)^ | 0.001^(f)^ | 0.096 | 1.894 | 0.0014 | 2.39 | 0.05 | 0.00003 | 3.73 |
|  | Pilot scale | 176^(i)^ | 10.8^(i)^ | 0.25^(i)^ | 0.573 | 12.787 | 0.349 | 14.33 | 0.32 | 0.00872 | 25.88 |
| **HSSF** | Full scale | 41.7^(f,h)^ | 3.09^(f,h)^ | 0.108^(d,f,h)^ | 0.169 | 4.541 | 0.187 | 4.21 | 0.11 | 0.00468 | 8.78 |
|  |  | 99.2^(a,b,c,d,f,h)^ | 2.6^(a,b,c,d,f,h)^ | 0.186^(a,b,c,d,h)^ | 0.401 | 3.821 | 0.322 | 10.02 | 0.10 | 0.00805 | 15.1 |
|  | Pilot scale | 146^(i)^ | 4.9^(i)^ | 0.396^(i)^ | 0.590 | 7.200 | 0.686 | 14.75 | 0.18 | 0.01714 | 24.9 |

The reported GHG (N_2_O-N, CO_2_-C, CH_4_-C) fluxes (as mg/m^2^ h) were based on references included in the review article of Mander et al. (2014).

**REFERENCES**

1. Mander Ü, Kuusemets V, Lõhmus K, Mauring T, Teiter S, Augustin J (2003) Nitrous oxide, dinitrogen, and methane emission in a subsurface flow constructed wetland. Water Sci. Technol. 48 (5): 135–142. <https://doi.org/10.2166/wst.2003.0301>
2. Mander Ü, Lõhmus K, Teiter S, Nurk K, Mauring T, Augustin J (2005a) Gaseous fluxes from subsurface flow constructed wetlands for wastewater treatment. J Environ Sci Health A 40 (6/7): 1215–1226. <https://doi.org/10.1081/ESE-200055662>
3. Mander Ü, Teiter S, Augustin J (2005b) Emission of greenhouse gases from constructed wetlands for wastewater treatment and from riparian buffer zones. Water Sci Technol 52 (10-11): 167–176. <https://doi.org/10.2166/wst.2005.0691>
4. Mander Ü, Lõhmus K, Teiter S, Mauring T, Nurk K, Augustin J (2008) Gaseous fluxes in the nitrogen and carbon budgets of subsurface flow constructed wetlands. Sci Total Environ 404: 343–353. <https://doi.org/10.1016/j.scitotenv.2008.03.014>
5. Mander Ü, Dotro G, Ebie Y, Towprayoon S, Chiemchaisri C, Nogueira SF, Jamsranjav B, Kasak K, Truu J, Tournebize J, Mitsch WJ (2014) Greenhouse gas emission in constructed wetlands for wastewater treatment: A review. Ecol Eng 66: 19–35. <http://dx.doi.org/10.1016/j.ecoleng.2013.12.006>
6. Søvik AK, Augustin J, Heikkinen K, Huttunen JT, Necki JM, Karjalainen SM, Kløve B, Liikanen A, Mander U, Puustinen M, Teiter S, Wachniew P (2006) Emission of the Greenhouse Gases Nitrous Oxide and Methane from Constructed Wetlands in Europe. J Environ Qual 35: 2360–2373. <https://doi.org/10.2134/jeq2006.0038>
7. Strom L, Lamppa A, Christensen TR (2006) Greenhouse gas emissions from a constructed wetland in southern Sweden. Wetlands Ecol Manage 15: 43–50. <https://doi.org/10.1007/s11273-006-9010-x>
8. Teiter S, Mander U (2005) Emission of N_2_O, N_2_, CH_4_, and CO_2_ from constructed wetlands for wastewater treatment and from riparian buffer zones. Ecol Eng 25: 528–541. <https://doi.org/10.1016/j.ecoleng.2005.07.011>
9. Van der Zaag AC, Gordon RJ, Burton DL, Jamieson RC, Stratton GW (2010) Greenhouse gas emissions from surface flow and subsurface flow constructed wetlands treating dairy wastewater. J Environ Qual 39 (2): 460– 471. <https://doi.org/10.2134/jeq2009.0166>

**Table S3** Meteorological data in the study area (period 1973-2020).

| **Month** | **Average precipitation height (mm/month)** | **Average temperature (^o^C)** | **Average wind speed**  **(knots)** | **Average relative humidity**  **(%)** | **Average evaporation ^(a)^**  **(mm/month)** | **Average evaporation ^(b)^ (mm/month)** | **Average evaporation ^(c)^**  **(mm/month)** |
| --- | --- | --- | --- | --- | --- | --- | --- |
| January | 106.0 | 9.86 | 8.75 | 73.3 | 85.5 | 74.1 | 78.8 |
| February | 85.5 | 10.2 | 9.89 | 72.59 | 85.3 | 111.7 | 73.6 |
| March | 66.9 | 12.13 | 8.31 | 70.6 | 113.7 | 172.9 | 94.4 |
| April | 36.8 | 15.65 | 6.23 | 68.31 | 136.8 | 215.9 | 114.2 |
| May | 13.8 | 20.13 | 5.74 | 65.64 | 171.2 | 281.6 | 151.6 |
| June | 1.5 | 24.78 | 6.56 | 59.64 | 197.9 | 278.9 | 191.3 |
| July | 0.6 | 27.07 | 8.94 | 56.21 | 209.2 | 285.2 | 224.1 |
| August | 0.1 | 26.81 | 9.03 | 57.78 | 195.4 | 279.4 | 219.0 |
| September | 13.2 | 23.16 | 7.54 | 62.81 | 156.7 | 183.2 | 173.2 |
| October | 27.5 | 18.6 | 7.51 | 70.31 | 128.3 | 12.1 | 133.8 |
| November | 77.0 | 14.27 | 7.48 | 74.6 | 96.1 | 69.6 | 97.9 |
| December | 118.3 | 11.23 | 8.47 | 73.88 | 85.7 | 61.7 | 85.1 |
| **Total** | **547.2** |  |  |  | **1661.8** | **2026.4** | **1637.0** |

^(a)^calculation with Equation Blanney-Criddle (1950); ^(b)^calculation with Equation Lamoreaux/Kohler (1962); ^(c)^calculation with Equation Penman/Linacre (1977)

**Table S4** Literature data on the percentage removal of COD, BOD and NH_4_-N in different aerobic biological Landfill Leachates Treatment Plants.

| **Technology** | **COD**  **Removal**  **(%)** | **ΒOD**  **Removal**  **(%)** | **ΝΗ_4_-Ν** **Removal**  **(%)** | **Reference** |
| --- | --- | --- | --- | --- |
| Activated sludge process | 55 ± 12 | 84 ± 15 | 94 ± 12 | Current study |
| Activated sludge process | 50 | 61 | 75 | Hoilijoki et al. 2000 |
| Activated sludge process | 60 | 90 | 98 | Tsompanoglou et al. 2023 |
| Aerobic ponds | 50 | 64 | 77 | Frascari et al. 2004 |
| Sequencing batch reactor | 76 | 84 | 65 | Neczaj et al. 2005 |
| Rotating biological contactors | 38 | 80 | 98 | Torretta et al. 2017 |
| Moving bed biofilm reactor | 60-81 | - | 92-95 | Loukidou and Zouboulis 2001 |
| Membrane bioreactor | 79 | 99 | 60 | Visvanathan et al. 2007 |

**Table S5** Parameters used for the design of horizontal subsurface flow constructed wetlands (HSSF) and surface flow constructed wetlands (FWS).

| **Design parameters** | **BOD** | **NH_4_-N** | **NO_3_-N** |
| --- | --- | --- | --- |
| ***HSSF*** | | | |
| Q (m^3^/d) | 27 | 27 | 27 |
| C,_in_ (mg/L) | 60 | 35 | 80 |
| C* (mg/L) | 10 | 0 | 0 |
| K_20_ | 25 | 11.4 | 41.8 |
| θ | 0.981 | 1.014 | 1.0 |
| P | 3 | 6 | 1 |
| T (^ο^C) | 20 | 20 | 20 |
| C,_out_ | 30 | 20 | 20 |
| Safety factor | 1.78 | 1.76 | 1.0 |
| ***FWS*** | | | |
| Q (m^3^/d) | 21.2 | 21.2 | 21.2 |
| C,_in_ (mg/L) | 30 | 20 | 20 |
| C* (mg/L) | 2 | 0,1 | 0 |
| K_20_ | 33 | 14.1 | 26.5 |
| θ | 0.985 | 1.014 | 1.102 |
| P | 1 | 3 | 3 |
| T (^ο^C) | 20 | 20 | 20 |
| C,_out_ | 20 | 10 | 10 |
| Safety factor | 1.78 | 1.76 | 1.0 |

**Table S6** Design characteristics of the constructed wetlands.

| **Design parameters** |  |
| --- | --- |
| *HSSF* | |
| Total area (m^2^) | 1,148 |
| Number of CWs | 2 |
| Area of each CW (m^2^) | 574 |
| Length/width ratio | 3:1 |
| Length (m) | 41 |
| Width (m) | 14 |
| Bed depth (m) | 0.5 |
| Area perpendicular to the flow (m^2^) | 7 |
| Organic loading rate per flow surface ((g/m^2^)/d) | 128 |
| Hydraulic residence time (d) | 7.4 |
| *FWS* | |
| Total area (m^2^) | 925 |
| Number of CWs | 2 |
| Area of each CW (m^2^) | 463 |
| Length/width ratio | 3:1 |
| Length (m) | 37 |
| Width (m) | 12.5 |
| Bed depth (m) | 0.3 |
| Hydraulic residence time (d) | 8.4 |

**Table S7** Daily flowrate of treated leachate from constructed wetlands for different months of the year.

| **Month** | **Outflow of constructed wetlands**  **(m^3^/day)** |
| --- | --- |
| January | 33.8 |
| February | 31.8 |
| March | 28.2 |
| April | 19.0 |
| May | 12.2 |
| June | 0.0 |
| July | 0.0 |
| August | 0.0 |
| September | 13.0 |
| October | 20.8 |
| November | 30.8 |
| December | 34.3 |

**Table S8** Water balance for the determination of the characteristics of evaporation ponds (total surface area of ponds: 4,440 m^2^).

| **Month** | **Monthly leachates flowrate (m^3^/month)** | **Monthly rain inflows (m^3^/month)** | **Monthly evaporation outflows (m^3^/month)** | **Monthly evaporation from Evaporator (m^3^/month)** | **Net volume of water in ponds (m^3^)** | **Water depth (m)** |
| --- | --- | --- | --- | --- | --- | --- |
| January | 1049 | 471 | 350 | 285.2 | 2,537 | 0.6 |
| February | 889 | 380 | 327 | 257.6 | 3,221 | 0.7 |
| March | 875 | 297 | 419 | 285.2 | 3,689 | 0.8 |
| April | 571 | 163 | 507 | 276.0 | 3,640 | 0.8 |
| May | 377 | 61 | 673 | 285.2 | 3,120 | 0.7 |
| June | 0 | 7 | 849 | 0.0 | 2,278 | 0.5 |
| July | 0 | 3 | 995 | 0.0 | 1,285 | 0.3 |
| August | 0 | 0 | 972 | 0.0 | 313 | 0.1 |
| September | 389 | 59 | 769 | 0.0 | 0 | 0.0 |
| October | 643 | 122 | 594 | 0.0 | 171 | 0.0 |
| November | 925 | 342 | 435 | 276.0 | 728 | 0.2 |
| December | 1063 | 525 | 378 | 285.2 | 1,653 | 0.4 |
| **Total** | **6781** | **2430** | **7268** | **1950** |  |  |

**Table S9** GHG emissions from public electricity and heat production (produced by liquid, solid and gaseous fuels) and GHG emission intensity for 2019.

|  | **GHG emissions**  **(in Mt)** | **GHG emission intensity**  **(in Kg/kWh)** |
| --- | --- | --- |
| **CO_2_** | 27.24 | 0.599 |
| **CH_4_** | 0.41^.^10^-3^ | 0.901^.^10^-5^ |
| **N_2_O** | 0.25^.^10^-3^ | 0.549^.^10^-5^ |

(Data of GHG emissions (in Mt) produced by liquid, solid and gaseous fuels was retrieved by Ministry of Environment and Energy (2021))

1. **Supplementary Figures**

**Fig. S1** Average monthly concentrations of COD of the incoming (raw) leachates in the studied Landfill Leachates Treatment Plant

**Fig. S2** Average monthly concentrations of NH_4_-N of the incoming (raw) leachates in the studied Landfill Leachates Treatment Plant

**Fig. S3** Monthly percentage removals of COD and BOD in the studied Landfill Leachates Treatment Plant for the period 24.2.2020 – 23.2.2021.

**Fig. S4** Monthly percentage removals of NH_4_-N in the studied Landfill Leachates Treatment Plant for the period 24.2.2020 – 23.2.2021.


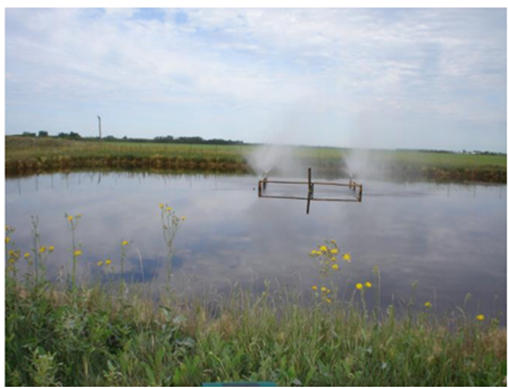


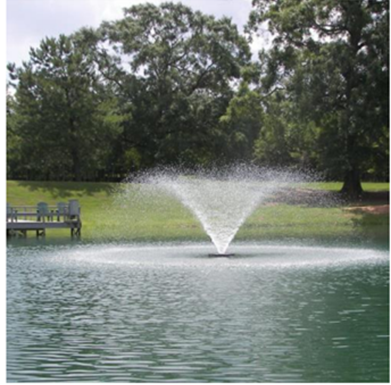


(a)

(b)

**Fig. S5** Devices for enhancing the evaporation in open ponds. System with spray nozzles (a), type Kasco VFX fountain evaporator (b).
